# Culturable fungal endophyte communities of primary successional plants on Mount St. Helens, WA, USA

**DOI:** 10.1186/s12862-022-01974-2

**Published:** 2022-02-15

**Authors:** Emily R. Wolfe, Robyn Dove, Cassandra Webster, Daniel J. Ballhorn

**Affiliations:** Department of Biology, Portland State University, PO Box 751, Portland, OR 97201 USA

**Keywords:** Plant–microbe interactions, Ecosystem processes, Microbe-microbe interactions, Fungi, Bacteria

## Abstract

**Background:**

While a considerable amount of research has explored plant community composition in primary successional systems, little is known about the microbial communities inhabiting these pioneer plant species. Fungal endophytes are ubiquitous within plants, and may play major roles in early successional ecosystems. Specifically, endophytes have been shown to affect successional processes, as well as alter host stress tolerance and litter decomposition dynamics—both of which are important components in harsh environments where soil organic matter is still scarce.

**Results:**

To determine possible contributions of fungal endophytes to plant colonization patterns, we surveyed six of the most common woody species on the Pumice Plain of Mount St. Helens (WA, USA; Lawetlat'la in the Cowlitz language; created during the 1980 eruption)—a model primary successional ecosystem—and found low colonization rates (< 15%), low species richness, and low diversity. Furthermore, while endophyte community composition did differ among woody species, we found only marginal evidence of temporal changes in community composition over a single field season (July–September).

**Conclusions:**

Our results indicate that even after a post-eruption period of 40 years, foliar endophyte communities still seem to be in the early stages of community development, and that the dominant pioneer riparian species Sitka alder (*Alnus viridis* ssp. *sinuata*) and Sitka willow (*Salix sitchensis*) may be serving as important microbial reservoirs for incoming plant colonizers.

**Supplementary Information:**

The online version contains supplementary material available at 10.1186/s12862-022-01974-2.

## Background

The Pumice Plain of Mount St. Helens (*Lawetlat'la* in the Cowlitz language) has served as a model for studying primary succession since its catastrophic eruption in 1980 [1, 2]. Old growth forest was obliterated and replaced with a 20 km^2^ layer of sterile pumice up to 200 m deep, creating a landscape that resembled the surface of the moon. A few years following the eruption, del Moral [3] observed just 3% vegetation cover on an exposed ridge affected by the pyroclastic flows, and by 1995, Lawrence and Ripple [4] estimated that vegetation cover on the Pumice Plain remained at 0–10% cover, with some areas of 11–20% cover. Even after 40 years following the eruption, the area remains largely barren—especially of woody species—except for sparse, stunted conifers and thickets of shrubs along riparian corridors [2, 5]. While there have been many studies of primary succession of vascular plants [6–9] and even their associated mycorrhizae [10–12], virtually no information exists on the diversity, temporal variation, and turnover of foliar endophyte communities colonizing these plants.

Endophytes are ubiquitous plant symbionts most simply defined as microbes spending the majority of their lifecycles living asymptomatically within host plant tissues [13, 14]. Fungal endophytes specifically have been most extensively studied in agricultural grass hosts, but have also been reported in important forest trees such as red alder [15, 16], maple [17, 18], oak [19, 20], and various conifers [21, 22]. Like other types of symbionts—e.g. root-associated mycorrhizal fungi or nitrogen-fixing *Frankia* and rhizobia—foliar endophytes can play critical roles in host plant fitness by affecting disease or pest resistance [23], drought tolerance [24], and overall competitive ability [25]. In addition to direct effects on host plants, foliar endophytes are also capable of altering plant community assembly [26, 27] and afterlife effects (reviewed in Wolfe and Ballhorn [28]) that can eventually affect ecosystem processes and nutrient cycling. These host–endophyte dynamics are especially important to consider in early successional ecosystems where effects may be disproportionate due to harsh environmental conditions, limited propagules, and sparse host plant communities.

Primary successional ecosystems are characterized by barren substrate produced by natural disasters or climate change, such as volcanic eruptions or receding glaciers [29, 30]. These ecosystems are frequently nutrient-limited, and may take centuries to develop into forests [31]. In these sparse environments, fewer propagules exist for microbial colonization of pioneering plant species [32]. Since microbial communities are critical to ecosystem function, a slow development thereof has significant implications for succession [33]. Dispersal limitation and environmental filters further reduce the pool of successful colonists in successional landscapes [34]. However, community assembly rules suggest that priority effects may alter successional trajectories of some species, which may contribute to changes in other communities or biogeochemical processes within an ecosystem. In a culture-based experiment that consisted of inoculating wooden disks with fungi and varying the initial colonizers to study how arrival order affected community composition and decomposition rates, Fukami et al. [35] emphasized the importance of assembly history in connecting community and ecosystem ecology. Given that the establishment and prevalence of plant–endophyte interactions are still widely unknown in new, anthropogenically-undisturbed ecosystems, we surveyed six major woody species on the Pumice Plain of Mount St. Helens and characterized the culturable fungal endophyte communities over a 3-month field season to answer the following questions: 1. How diverse are culturable foliar endophyte communities in this early successional ecosystem? 2. Do these communities exhibit host specificity? and 3. Do these endophyte communities show temporal variation over a growing season?

## Results

**Fig. 1:**
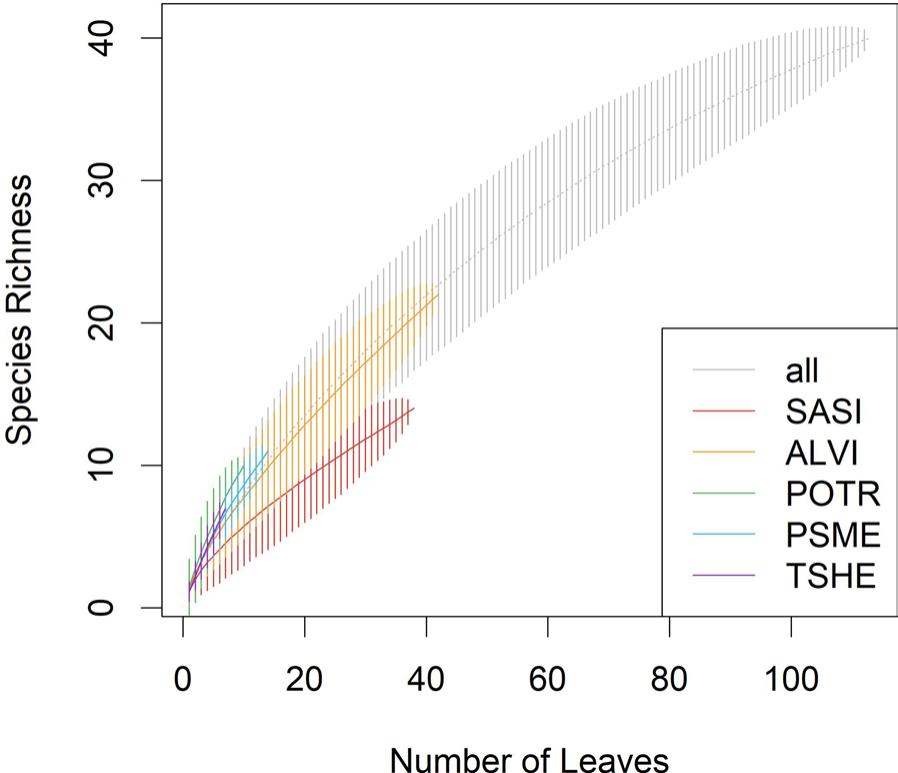
Species (i.e., OTU) accumulation curves indicating that sampling more leaves would have detected additional culturable taxa

We isolated foliar fungal endophytes from the six most abundant woody species on the Pumice Plain of Mount St. Helens throughout the 2017 summer field season, and found species-specific endophyte community composition despite overall low colonization rates. A total of 40 OTUs were isolated from 113 leaf samples (14.1% of total n = 801 leaves; 19.4% of surveyed trees), representing 18 families and 23 genera. The highest number of OTUs were isolated from Sitka alder (22), followed by Sitka willow (14), cottonwood (10), Douglas fir (11), western hemlock (7), and noble fir (1). All of the OTUs belonged to Ascomycota except for one member of Basidiomycota, and included a total of four classes: Dothidiomycetes (16 OTUs), Pezizomycetes (13 OTUs), Sordariomycetes (9 OTUs), Eurotiomycetes (1 OTU), and Agaricomycetes (1 OTU). Frequencies of isolation ranged from 45.13% (OTU01; *Arthrinium* sp.) to 0.885% for singletons (18 OTUs), averaging 3.12 ± 7.12% (mean ± SD) over all cultured OTUs. As anticipated given the limitations of culture-based techniques, the species accumulation curve showed that sampling more leaves would have revealed additional taxa, especially for conifer host species (Fig. 1).

**Fig. 2:**
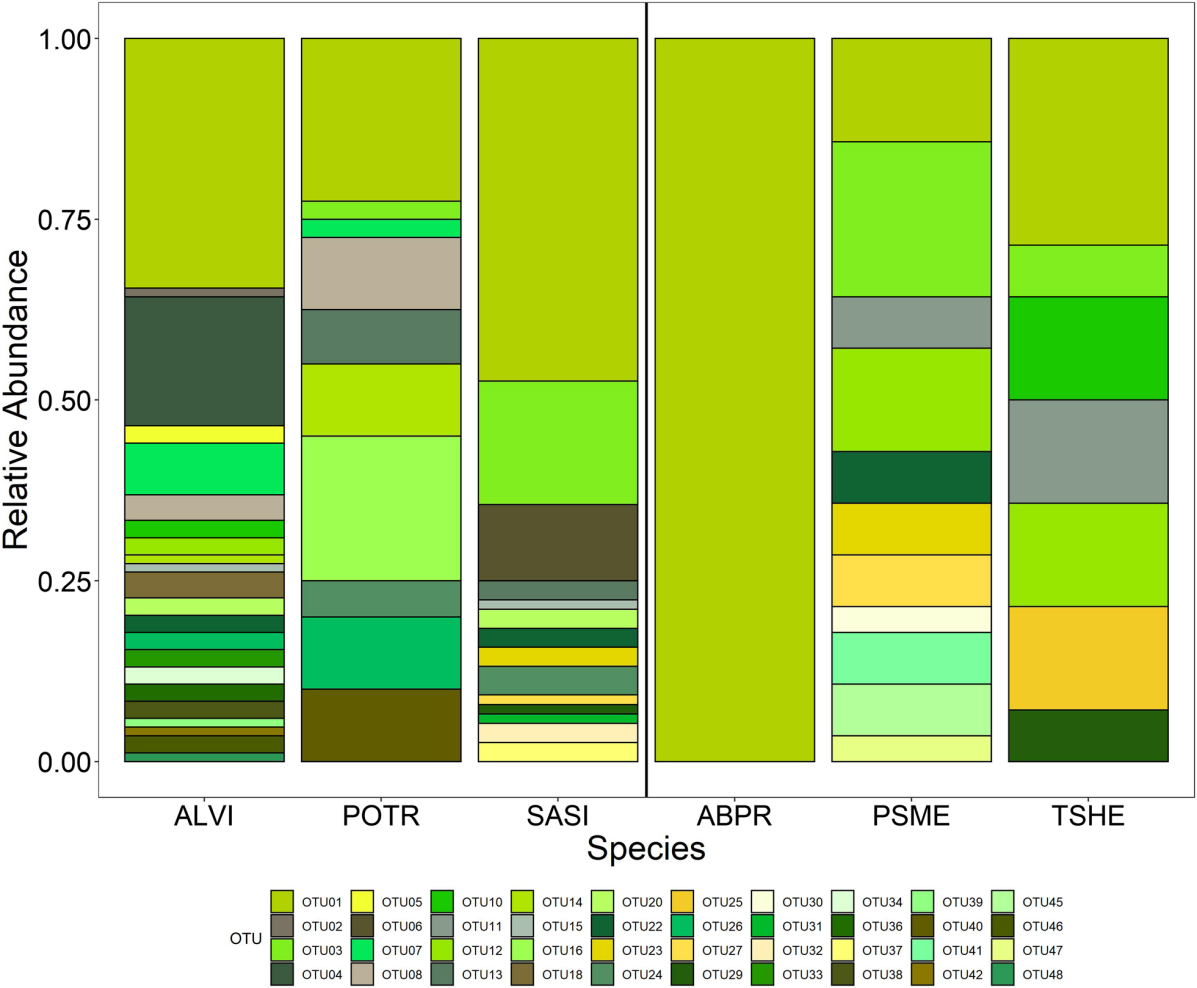
Relative abundance of culturable OTUs among the six prominent woody species colonizing the Pumice Plain

**Fig. 3:**
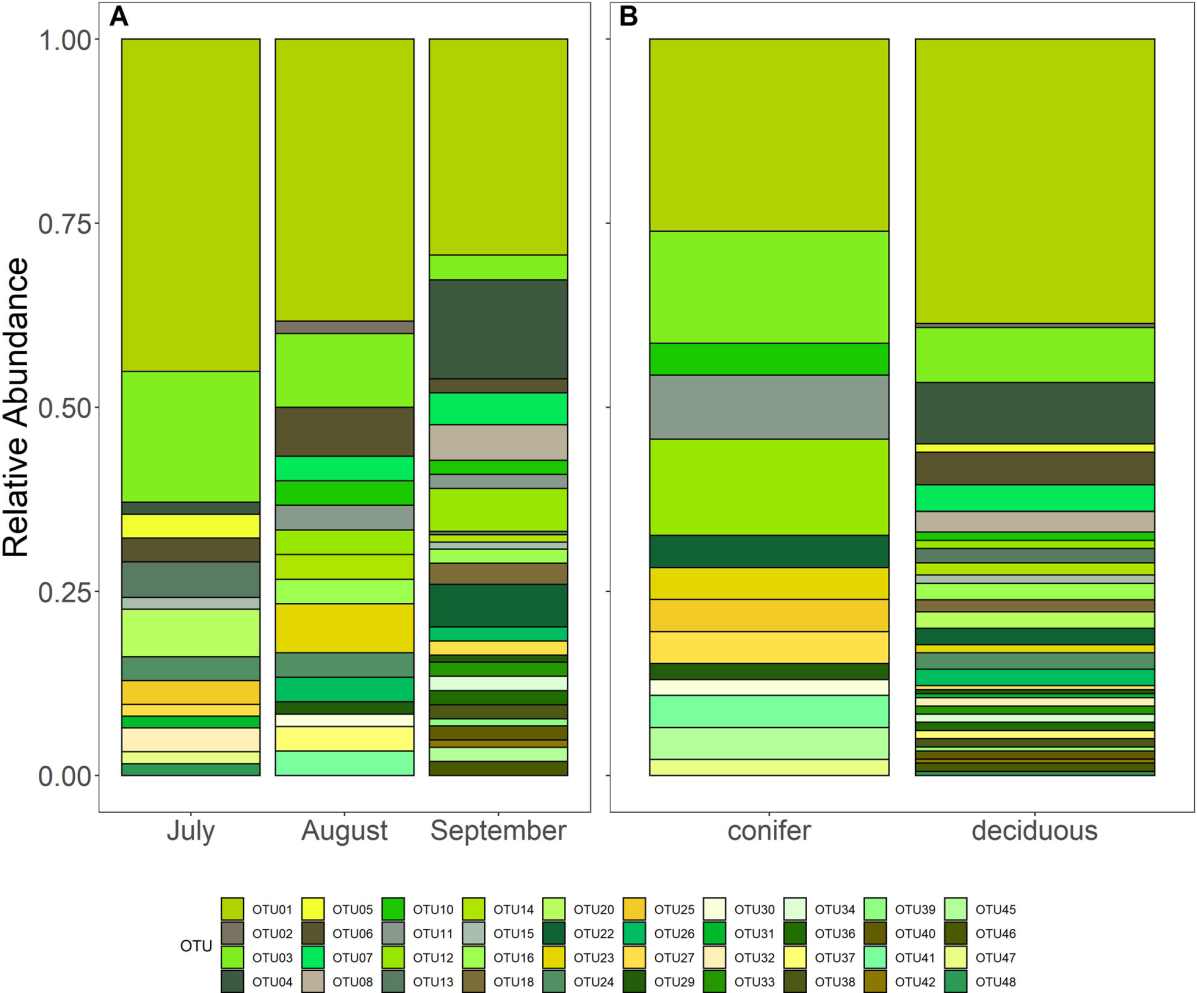
Relative abundance of culturable OTUs (**A**) over the 3-month field season, and **B** between host tree type

While the fungal endophyte community composition differed significantly among species (PERMANOVA, F_5,112_ = 1.7781, p = 0.004; Fig. 2), it did not vary over time (PERMANOVA, p = 0.087; Fig. 3A), and subsequently there was no significant interaction between host species and time (Additional file 1: Figure S1). Tree type (i.e. conifer vs. deciduous) however did have a significant effect on community composition (PERMANOVA, F_1,112_ = 1.9764, p = 0.035; Fig. 3B). The endophyte community composition also varied significantly between deciduous host species (PERMANOVA, F_2,89_ = 2.4337, p = 0.002), but only marginally over time (PERMANOVA, F_2,89_ = 1.4523, p = 0.099; Additional file 1: Figure S2). Overall OTU richness (1.248 ± 0.492) and Shannon diversity indices (0.166 ± 0.311) were low, and no significant differences existed either among species or over time for either the entire community or the deciduous subset. Over three-quarters of leaves yielded a single OTU, while the remaining leaves had at least two, including one cottonwood leaf (Site 62, harvested in September) that had the highest species richness of 4.

Only OTU16 (*Tricharina praecox*) was significantly associated with a particular host plant species (cottonwood; indicator species analysis, IV = 0.447, p = 0.040), while OTU11 (unclassified Pleosporales sp.; IV = 0.295, p = 0.045) and OTU12 (*Sporormiella intermedia*; IV = 0.347, p = 0.035) were associated with conifer hosts. OTU04 (*Melanconis italica*) was almost exclusively isolated from samples collected in September (IV = 0.357, p = 0.045). Within the alder-willow subset, OTU03 (*Pseudoplectania episphagnum*) and OTU06 (Pyronemataceae sp.) were associated with Sitka willow as a host species (IV = 0.459, p = 0.005; IV = 0.324, p = 0.035, respectively), while OTU04 (*Melanconis italica*) was associated with Sitka alder hosts (IV = 0.463, p = 0.010).

## Discussion

Our study revealed that after nearly four decades, culturable foliar fungal endophytes have been slow to colonize major woody host species on the early primary successional Pumice Plain of Mount St. Helens. In contrast, vegetation communities stabilized on the nearby Plains of Abraham after just two decades [36]. Additionally, in red alder trees in the Portland Metro area and Tillamook State Forest (both in Oregon), we found significantly higher numbers of OTUs and a smaller proportion of singletons from over 95% of leaves sampled, compared to less than 15% of leaves sampled at the Mount St. Helens Pumice Plain [15]. In conifers throughout the Pacific Northwest, Carroll and Carroll [21] found that the percentage of infected needles ranged from 20–100% depending on the conifer host species, while we observed an overall colonization rate of 14.1% on the Pumice Plain. This colonization rate is consistent with the colonization rate in needles of *Pinus taeda* seedlings (14.0%; [37]). Overall, on the Pumice Plain we observed colonization rates that were considerably lower than those that have been recorded for both deciduous and coniferous trees in the region using similar or identical culture-based methods [15, 16, 21].

The low richness and diversity of the endophyte community are an order of magnitude smaller than values measured in nearby non-successional (i.e. significantly later stage) sites [15], suggesting there could be a sizeable lag between the establishment of host plants and eventual foliar colonists on the Pumice Plain. Similar patterns have been observed in root-associated symbionts (i.e. mycorrhizae, dark septate endophytes) at the forefront of a receding glacier [12, 38], as well as in mycorrhizal and *Alnus*-associated *Frankia* communities also on the Pumice Plain [39, 40], although soil microbial communities appear to be structured by different factors during early succession [41]. While plant community richness may have peaked in some areas of the Pumice Plain [42], plant cover is largely patchy and separated by stretches with inhospitable conditions. Conifers are especially sparse on the Pumice Plain, while substantial thickets of Sitka willow and alder are present along riparian corridors and within seeps, and clusters of the shrubs are interspersed throughout the upland areas. Propagules of endophytes may be present but unable to effectively disperse or establish, as was the case for early mycorrhizae just a few years following the eruption [40]. Consequently, given that the presence and proximity of host plants were important factors for foliar endophyte colonization of beachgrasses in coastal sand dunes [13], the infrequent distribution of host plants on the Pumice Plain compared to surrounding, less impacted areas likely contributed to the poverty of the culturable endophyte community. Additionally, microclimatic conditions and local soil chemical features play particularly significant roles in determining plant community establishment and composition on the Pumice Plain [42–44], and may act as additional filters for endophyte colonization.

Endophyte community compositions were host-specific and included several generalist taxa—a pattern consistent with other studies on oak, cypress, ash, and maple [45, 46]. The most commonly isolated OTU (*Arthrinium* sp.) was found in all six species, and members of the genus have been previously reported in the leaves and stems of *Alnus* sp. [47], *Salix* sp. [48], and *Populus* sp. [49], as well as in a variety of both temperate and tropical plants [50–53]. To our knowledge, we are the first to report *Arthrinium* sp. in Douglas fir, western hemlock, and noble fir, and *Pseudoplectania* sp. in Sitka willow. Notably, *Arthrinium* sp. was isolated from *Salix alba* infested with the woodwasp *Xiphydria* that disperses its fungal symbionts between trees [48], and both willow and cottonwood shrubs are affected by stem-boring, poplar-willow weevils on the Pumice Plain [54]. A number of taxa isolated from these particular weevils can also occur as generalist endophytes [55], and demonstrate a possible dispersal vector that should be further investigated with molecular techniques.

Surprisingly, we did not find a significant temporal effect on foliar endophyte community composition throughout the vegetation period, contrary to other studies [56, 57], including one that was conducted in the same geographical region [58]. We did obtain nearly four times as many cultures from deciduous compared to conifer hosts—reflecting the significant effect of tree type on community composition—but even the deciduous community that excluded conifers only marginally varied over the 3-month field season. This pattern was largely driven by the presence of OTU01 (*Arthrinium* sp.) in nearly every harvest for each host species and a considerable number of singletons. The three most abundant genera that were isolated are broadly classified as either saprotrophs or pathogens in the FUNGuild database [59]. Initial colonists also have significant priority effects on the trajectories of endophyte community composition [60], and may explain some of the shared colonization patterns on the Pumice Plain. However, culture-based methods are inherently more biased than culture-independent methods due to variation in surface-sterilization effectiveness, media used, and the low percentage of microbes that are capable of growing in culture. Consequently, overrepresentation of fast-growing, possibly aggressive competitors should not be unexpected, especially in conjunction with culture-based techniques.

Host plant age can also be an important factor determining endophyte presence and diversity [37]. The trees sampled in this study ranged between 1 and 3 m in height and likely were younger than trees sampled in comparable studies, but the true variation in ages and any effects it may have had on our results are unknown [15, 21, 37, 61]. In addition, leaf age can significantly affect the foliar endophyte community [62, 63], and may have influenced the taxa that we were able to isolate, at least in regard to the conifer hosts. Needles in particular were sampled near the ends of branches, and new growth that had not been colonized yet may have been overrepresented. Carroll and Carroll [21] also cite high elevation as a factor in affecting the quality of a plant host—particularly for *Tsuga heterophylla*, also sampled here—which may also apply to our field site (~ 1000 m a.s.l.). Still, the strikingly low diversity of endophytes at our study site across multiple tree species suggests slow succession of these microbial foliar communities.

## Conclusions

Our findings are an important first step in further characterizing the plant-associated microbial community of the Pumice Plain, a particularly well-studied primary model ecosystem, which has so far only included studies of mycorrhizae [10, 40] and *Frankia* symbionts [39, 64]. We found that culturable endophyte communities are dominated by saprotrophs and pathogens and overall show high species-specificity, although there are obvious biases and limitations of both culture-based techniques and Sanger sequencing. The endophyte communities we identified exhibited remarkably little diversity—in particular in comparison to relatively closely located control sites—and no statistically significant temporal variation, which may be a function of both uneven sampling depth due to limitations in the field and culture-based methods. Regardless, dominant pioneer riparian species like Sitka willow and alder are likely serving as important microbial reservoirs and facilitators for subsequent plant colonization. Primary successional systems like the Pumice Plain are unique models that allow critical insight into how communities and ecosystems reestablish after catastrophic destruction. While mock communities and experimental manipulation can help clarify the underlying mechanisms within, and effects of, plant–microbe interactions, the well-documented eruption and initial stages of recovery on Mount St. Helens are especially crucial for observing the way that plant–microbe interactions develop after catastrophic events.

## Methods

**Fig. 4:**
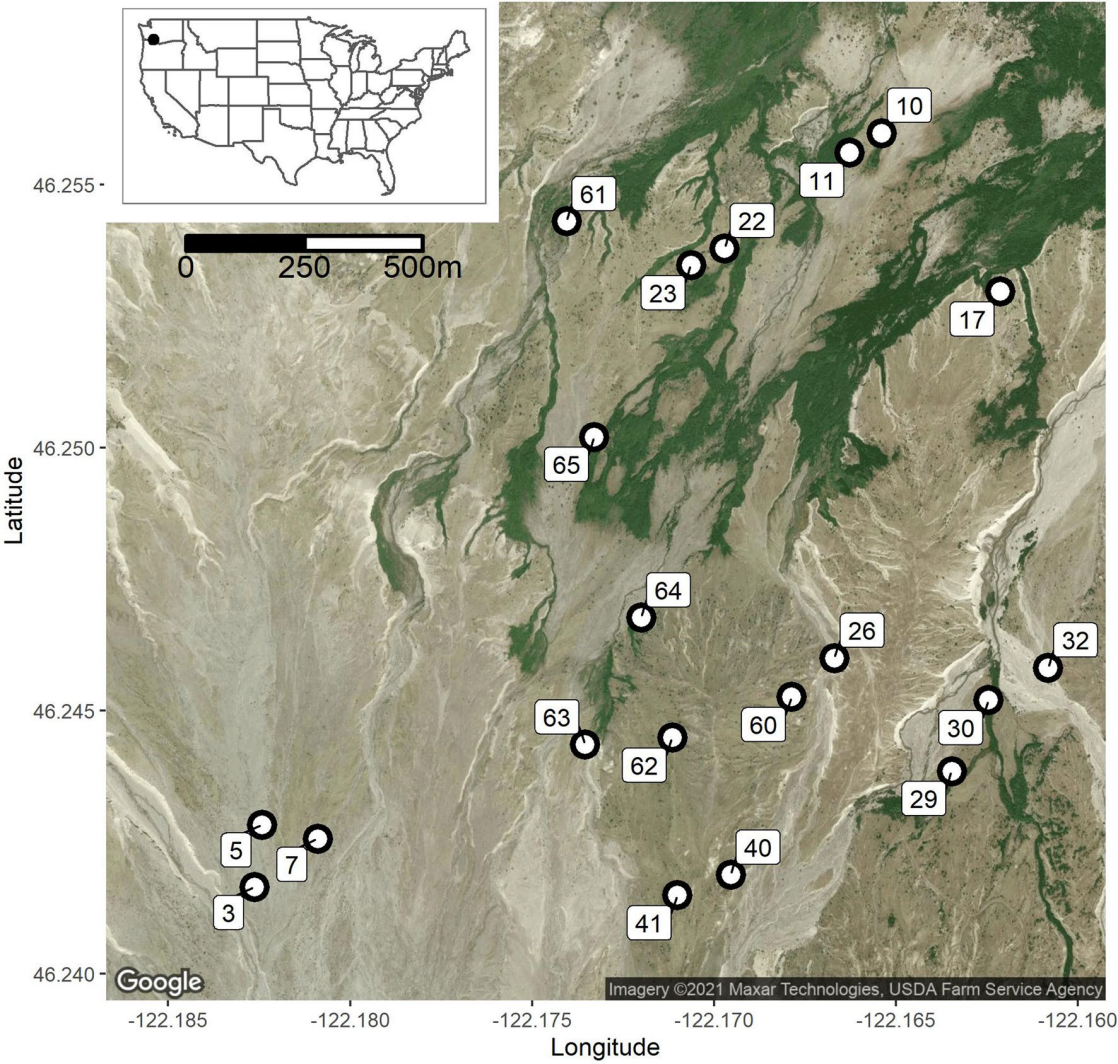
Map of the twenty sampling sites on the primary successional Pumice Plain of Mount St. Helens, WA, USA

Between July and September 2017, we sampled six common woody species found on the Pumice Plain of Mount St. Helens, WA [65]: Sitka alder, Sitka willow, cottonwood, Douglas fir, noble fir, and western hemlock (Table 1). Sites were identified by traversing previously-established horizontal transects [66, 67] and collecting GPS coordinates for over 300 trees. Using GPS coordinates and the R package Imap, trees less than 100 m from one another were clustered into sites; however, any prospective sites with fewer than four of the six species of interest present were excluded from final consideration. Twenty sampling sites (n = 371 trees) were identified based on the number of species, whether there were more than two individuals of a given species present (maximum of 3 were sampled per species per site), and if the sites were reasonably accessible (Fig. 4). Except in the case of no remaining leaves, the same trees were sampled every 30 days during the 3-month field season (July–September). Either three deciduous leaves or 5–10 cm branch pieces (conifers) were randomly collected from each tree using gloves sterilized with 95% ethanol. Samples were collected and transported to the lab in a cooler (< 15 ℃) on the same day, which contributed to practical limitations for sample collection and the effective sample size. Leaves were stored at 4 ℃ for less than 48 h before surface-sterilization. The following minor modifications were made to the methods detailed in Wolfe et al. [15] to accommodate bulk surface-sterilization of leaves: custom sheets of mesh sleeves were constructed from window-screen and sterilized prior to use, and then used to transfer leaves between washes in sterilized polypropylene bins. Sheets of leaves were air-dried in a laminar-flow hood and then processed aseptically onto malt extract agar (MEA) plates. We verified the efficacy of these modifications by re-sampling previously studied red alder (*Alnus rubra*) trees in the Portland Metropolitan area and finding similar colonization rates as Wolfe et al. [15].

**Table 1:** Dominant woody species of the Pumice Plain on Mount St. Helens, WA, including the three most common species for both riparian and upland areas

Scientific name	Common name	Abbreviation
*Alnus viridis* ssp. *sinuata*	Sitka alder	ALVI
*Salix sitchensis*	Sitka willow	SASI
*Populus balsamifera* ssp. *trichocarpa*	Black cottonwood	POTR
*Pseudotsuga menziesii*	Douglas fir	PSME
*Abies procera*	Noble fir	ABPR
*Tsuga heterophylla*	Western hemlock	TSHE

Plates were checked every 3 days for fungal growth, and any new isolates were transferred to new MEA plates until axenic. While isolates were initially differentiated by morphology, all new growth was transferred to new MEA plates for verification through Sanger sequencing. Fungal isolates from MEA plates were processed with the Sigma Tissue Extract-N-Amp Kit (St. Louis, MO). We used primers ITS1F and ITS4 to amplify DNA with the following thermocycler settings: 34 cycles of 94 ℃, 50 ℃, and 72 ℃ for 1 min each, followed by a final extension at 72 ℃ for 10 min. Products were checked with gel electrophoresis before being sent to Function Biosciences (Milwaukee, WI) for Sanger sequencing. Raw sequencing data were manually inspected and cleaned in Geneious 10.2.3. The output was then aligned with MAFFT on XSEDE v7.427 [68, 69] and imported into mothur v.1.42.3 [70] to generate both putative taxonomic assignments through UNITE [71] and operational taxonomic unit (OTU) tables (sequence submission pending at GenBank). OTUs were clustered at 99% similarity [72] using the furthest-neighbor algorithm [61].

Data were analyzed with R v. 3.6.3 with vegan, car, and indicspecies [73, 74], and abundance figures were generated with phyloseq [75], while the map in Fig. 4 was created with ggmap [76]. Differences in community compositions were first tested for homogenous dispersion within and among groups (betadisper in vegan; Bray–Curtis), and then compared with permutational multivariate analysis of variance (PERMANOVA; adonis2; Bray–Curtis distance, and 999 permutations) for all host species, tree type (conifer vs. deciduous), and collection month (July, August, or September). Since over three-quarters of the isolates came from deciduous hosts, we created a subset of the community for those species (alder, willow, and cottonwood), and used PERMANOVA tests to compare differences in species composition and collection month after verifying dispersion was homogeneous within and among all groupings (i.e., by species or over time). Non-metric multidimensional scaling ordinations (Bray–Curtis distances, k = 3 determined by screeplot) were used to visualize community compositions. Richness and Shannon diversity indices were calculated using vegan. If assumptions could not be met, nonparametric Kruskal–Wallis tests were used instead.

## Supplementary Information

**Additional file 1: Figure S1.** NMDS ordination of all host species and all harvest dates. **Figure S2.** NMDS ordination of deciduous hosts only (Sitka alder, Sitka willow, and cottonwood) and all harvest dates.

## Data Availability

The raw data are available in the following GitHub repository: https://github.com/emwolfe/MSH_endophytes.

## Abbreviations

*A. procera*/ABPR: Noble fir
*A. viridis* or *A. viridis* ssp*. sinuata*/ALVI: Sitka alder
MEA: Malt extract agar
OUT: Operational taxonomic unit
*P. menziesii*/PSME: Douglas fir
PERMANOVA: Permutational multivariate analysis of variance
*S. sitchensis*/SASI: Sitka willow
*T. heterophylla*/TSHE: Western hemlock
