# Supplementary material for: Culturable fungal endophyte communities of primary successional plants on Mount St. Helens, WA, USA
Source: BMC Ecol Evol. 2022 Feb 15;22:18. doi: 10.1186/s12862-022-01974-2 (PMC8845407; doi:10.1186/s12862-022-01974-2)

Supplemental Figure 1. NMDS ordination of all host species and all harvest dates.
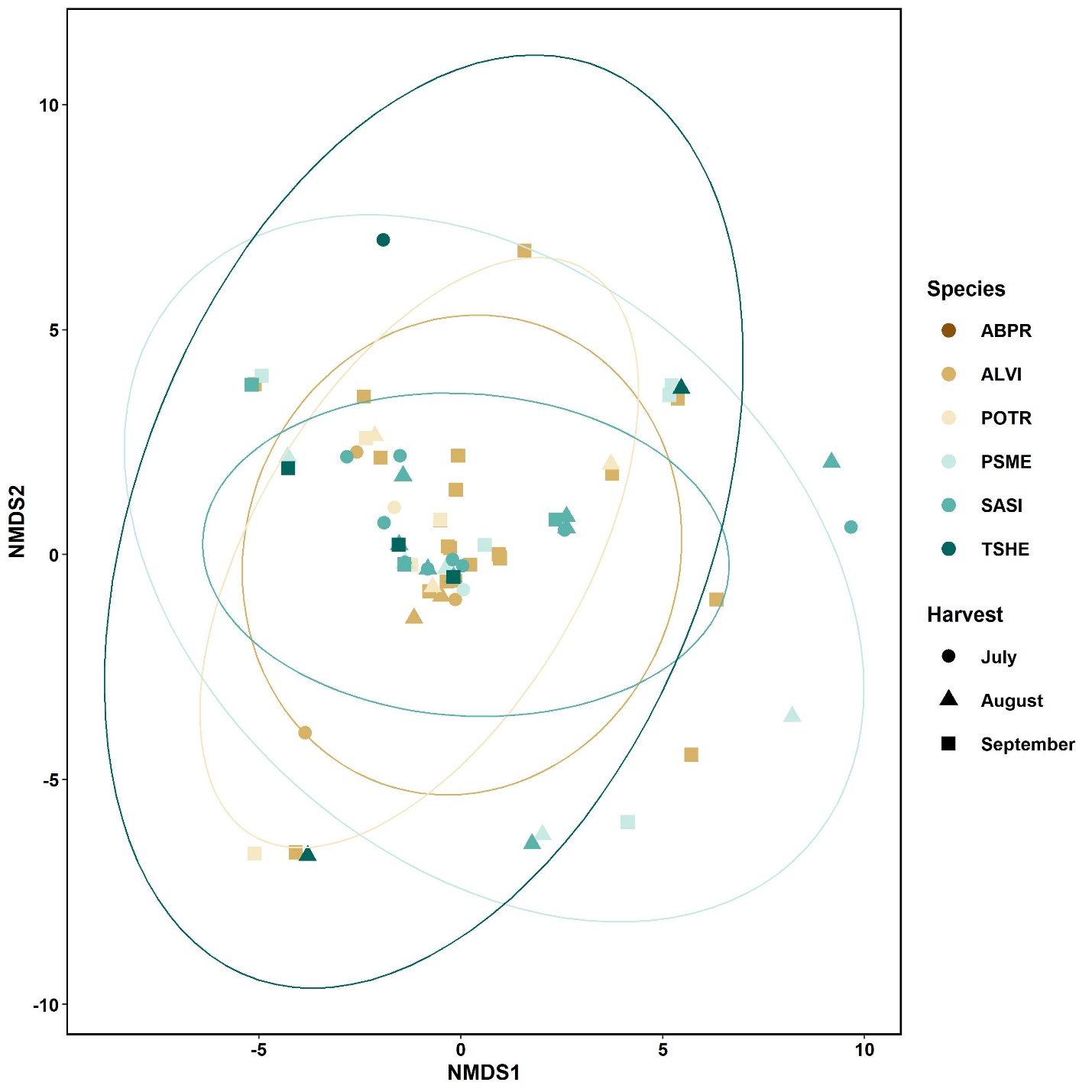


Supplemental Figure 2. NMDS ordination of deciduous hosts only (Sitka alder, Sitka willow, and cottonwood) and all harvest dates.


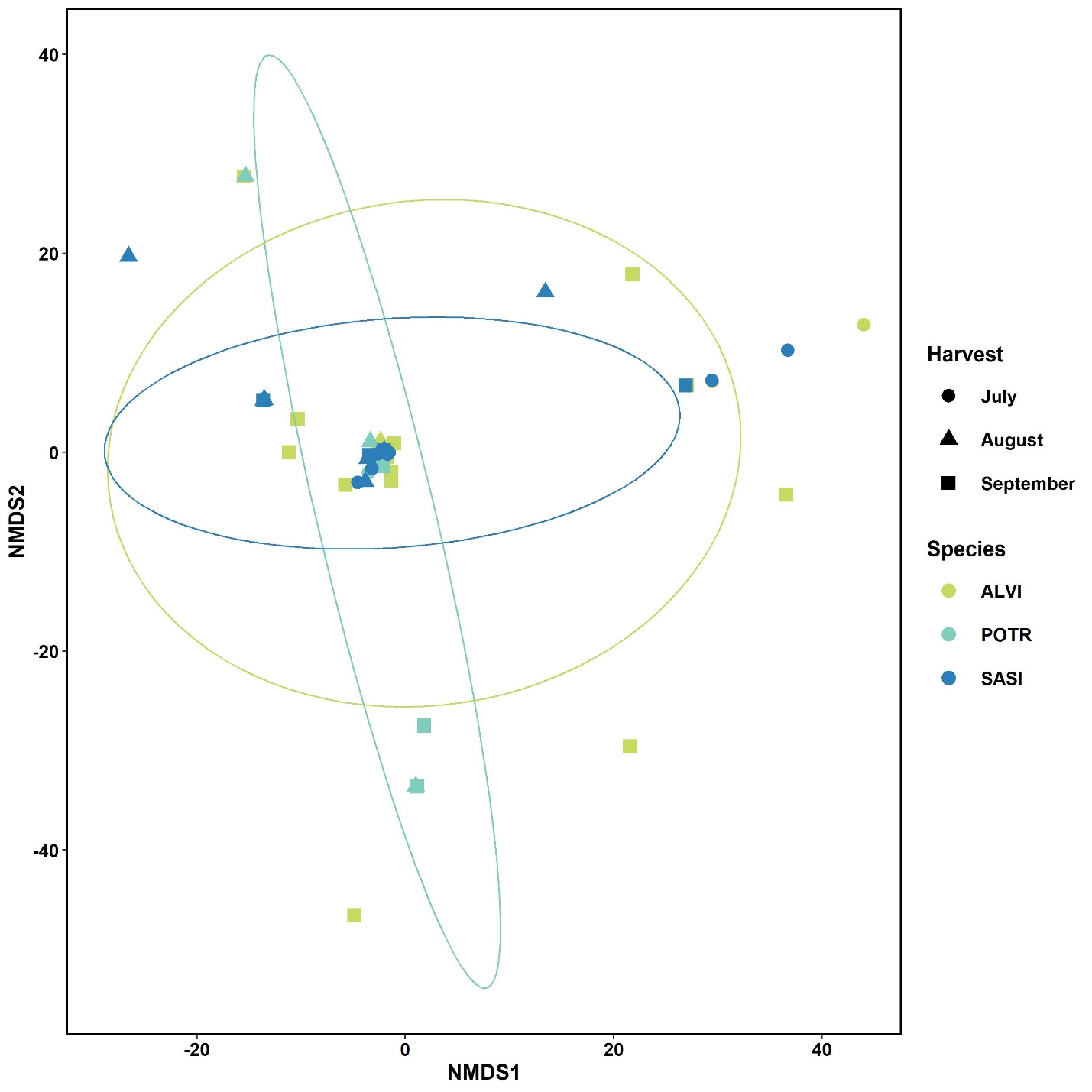

Supplement: Supplementary file 1 — Additional file 1: Figure S1. NMDS ordination of all host species and all harvest dates. Figure S2. NMDS ordination of deciduous hosts only (Sitka alder, Sitka willow, and cottonwood) and all harvest dates. [file 12862_2022_1974_MOESM1_ESM.docx]
